# Detection of the oil-producing microalga *Botryococcus braunii* in natural freshwater environments by targeting the hydrocarbon biosynthesis gene *SSL-3*

**DOI:** 10.1038/s41598-019-53619-y

**Published:** 2019-11-18

**Authors:** Kotaro Hirano, Takuya Hara, Ardianor, Rudy Agung Nugroho, Hendrik Segah, Naru Takayama, Gumiri Sulmin, Yukio Komai, Shigeru Okada, Koji Kawamura

**Affiliations:** 1 Department of Environmental Engineering, Osaka Institute of Technology, Osaka, Japan; 2 University of Palangka Raya, Palangkaraya, Indonesia; 3 Faculty of Mathematics and Natural Sciences, Mulawarman University, Samarinda, Indonesia; 4 Graduate School of Agricultural and Life Sciences, The University of Tokyo, Tokyo, Japan

**Keywords:** Water microbiology, Microbial ecology

## Abstract

The green microalga *Botryococcus braunii* produces hydrocarbon oils at 25–75% of its dry weight and is a promising source of biofuel feedstock. Few studies have examined this species’ ecology in natural habitats, and few wild genetic resources have been collected due to difficulties caused by its low abundance in nature. This study aimed to develop a real-time PCR assay for specific detection and quantification of this alga in natural environments and to quantify spatiotemporal variations of wild *B*. *braunii* populations in a tropical pond. We designed PCR primers toward the hydrocarbon biosynthesis gene *SSL-3* and examined amplification specificity and PCR efficiency with 70 wild strains newly isolated from various environments. The results demonstrated that this PCR assay specifically amplified *B*. *braunii* DNA, especially that of B-race strains, and can be widely used to detect wild *B*. *braunii* strains in temperate and tropical habitats. Field-testing in a tropical pond suggested a diurnal change in the abundance of *B*. *braunii* in surface water and found *B*. *braunii* not only in surface water, but also at 1–1.5 m deep and in bottom sediments. This method can contribute to efficient genetic resource exploitations and may also help elucidate the unknown ecology of *B*. *braunii*.

## Introduction

**Figure 1:**
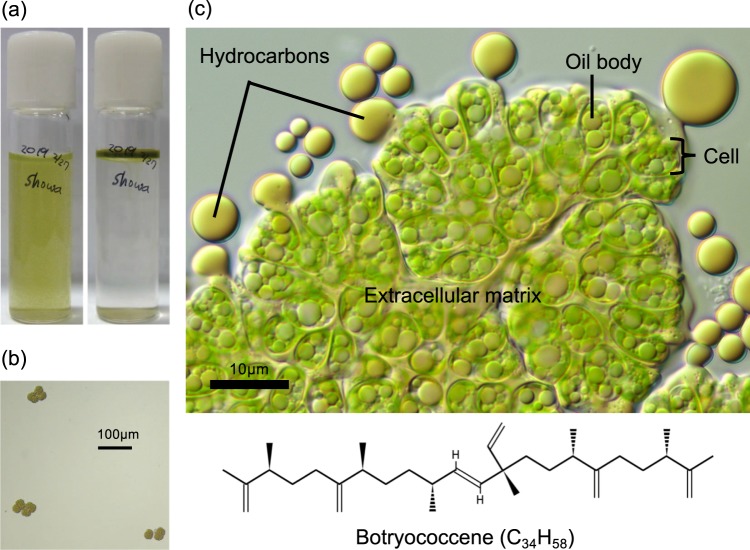
Characteristics of *Botryococcus braunii* strain Showa (race B) colonies. *Botryococcus braunii* is a colonial green alga. The Showa strain produces C_n_H_2n-10_ (n = 30–34) hydrocarbons called botryococcenes. Botryococcenes are synthesized inside the cells of the colony and are observed as intracellular oil bodies that are secreted into the colony extracellular matrix where the majority of the hydrocarbons are stored. (**a**) A 10-mL glass tube culture of Showa: Left, mixed gently; Right, left undisturbed overnight, where the colonies floated upward. (**b**) Microscopic view of the Showa colonies. (**c**) The Showa colony was flattened by cover glass, and hydrocarbons exuded from extracellular matrices.

Microalgae are a promising renewable feedstock for biofuel production with the potential to serve as a practical alternative to petroleum-based transportation fuels^1–3^. Microalgal oils produced by major oleaginous microalgae, such as *Chlorella* spp. and diatoms, are triacylglycerols, and few species are known to produce hydrocarbons^4^. Hydrocarbons are preferred over triacylglycerols because of their high energy density and compatibility with existing petroleum infrastructure^5^. The colonial microalga *Botryococcus braunii* produces hydrocarbon oils at 25–75% of its dry weight and is one of the most promising candidates for biofuel feedstock production^1^. The hydrocarbons are mainly stored in the extracellular space (Fig. 1), is contrast to most other oleaginous microalgae which accumulate lipids in the cytoplasm^6^. A catalytic hydrocracking converts the hydrocarbon oils into transport fuels such as gasoline, kerosene, and diesel^7,8^. Fossils of *B*. *braunii* have been identified in organic remains of oil shales and petroleum source rocks^9^, and their hydrocarbon oils are shown to be a major component of crude oils^10,11^, indicating the noticeable contribution of this alga to petroleum generation. Despite these remarkable characteristics of *B*. *braunii*, it has not yet been implemented in practical use for biofuel feedstock production. This is mainly due to its relatively slow growth rate, which makes mass cultivation in open ponds difficult to control due to competition with fast-growing microalgae^12,13^. The fastest growth rate in *B*. *braunii* has been recorded by a wild strain, Showa, with a doubling time of 1.4 days^14^. Because *B*. *braunii* is widespread in freshwater and brackish lakes, reservoirs, or ponds from temperate to tropic environments^13^, it should be possible to find a novel strain that grows faster than Showa, but few studies have investigated wild genetic resources. Furthermore, there have been several reports of natural blooms of this species^15–19^, but the underlying mechanisms remain largely unknown. Elucidating the ecological and environmental factors associated with the natural blooming, including the presence of bacterial symbionts^20^ and density of competitors or natural enemies, would provide helpful information for the development of an open-pond *B*. *braunii* cultivation system. However, the density of *B*. *braunii* in natural environments is normally very low (10–10^2^ colonies per L), which complicates quantitative investigations. Furthermore, natural life cycles of the species, including sexual reproduction and dormancy, are completely unknown. We therefore might not identify this alga in natural environments if it changes morphology, into a single-cell, gamete, or zygote form, for example. Thus, to facilitate ecological studies of *B*. *braunii* in natural environments, this study aimed to develop a real-time PCR assay for specific detection and quantification of wild *B*. *braunii*.

Real-time PCR is a highly sensitive technique for detecting and quantifying target DNA molecules. Target gene selection is the most important factor for the specific detection of a target organism. We focused on a hydrocarbon biosynthetic gene, *squalene synthase-like protein 3* (*SSL-3*)^21^ to achieve the specific detection of *B*. *braunii* DNA. Three chemical races of *B*. *braunii* (A, B, and L) have been identified, and their classification depends on their hydrocarbon structures. Race A produces fatty acid-derived C_23_–C_33_ alkadienes and triene hydrocarbons. Races B and L produce isoprenoid-derived hydrocarbons; Race B produces triterpenoid hydrocarbons, C_30_–C_37_ botryococcenes, and C_31_–C_34_ methylated squalenes, while race L produces the tetraterpenoid hydrocarbon C_40_ lycopadiene^22^. Recently, a new class of strain tentatively termed race S was identified^23^, and these strains synthesize C_18_ epoxy-*n*-alkanes and C_20_ saturated *n*-alkanes. *SSL-3* encodes an enzyme that catalyses the final step of B-race hydrocarbon biosynthesis^21^ and is therefore expected to be useful for specific detection of *B*. *braunii*, especially of B-race strains. In fact, environmental studies suggested that botryococcenes are specific biomarkers of *B*. *braunii*^24,25^. The A- and B-race strains have generally higher contents of hydrocarbons compared to L-race strains, which contain only a few percent hydrocarbons. The B-race hydrocarbons are likely a more appropriate source for biofuels than the A-race hydrocarbons owing to their branched, unsaturated structures.

The objective of this study was to assess the applicability of a real-time PCR assay of the *SSL-3* gene for specific detection and quantification of *B*. *braunii* (race B) in natural environments, by examining (1) the efficiency of DNA extraction, (2) the amplification specificity of PCR, and (3) the applicability to wild strains. The chemically stable and physically resistant hydrocarbon matrix of *B*. *braunii* may reduce the efficiency of DNA extraction^26^. We therefore first assessed the efficiency of DNA extraction of our method. The PCR-based approach has a trade-off between specificity and generality. To reduce the risk of amplifying DNA from off-target organisms, it is necessary to design specific primers, but the use of highly specific primers may reduce the amplification efficiency of target organism DNA because of the possibility of genetic variation in primer-binding sequences. We therefore tested both the risk of off-target amplification and the wide applicability to genetically diverse wild strains isolated from temperate to tropical aquatic environments. Finally, (4) we show the results of our real-time PCR-based quantification of spatiotemporal variations of a wild *B*. *braunii* population in a tropical pond and discuss how to use our method for future ecological studies.

## Results and Discussion

### Standard curve generation with the Showa strain

**Figure 2:**
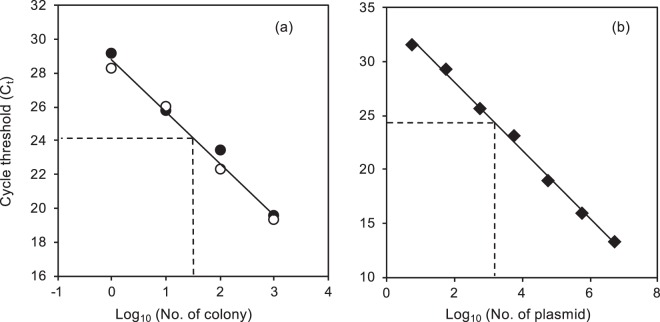
Standard curve of a real-time PCR assay for the *SSL-3* gene in *Botryococcus braunii*. Standard curves were generated by correlating Ct values with the Log_10_-transformed number of colonies and number of plasmids. (**a**) Standard curves generated from two series of standard samples with independent dilution and DNA extraction procedures (●, ○): y = −3.08 × +28.8 (R^2^ = 0.987, *P* < 0.001). (**b**) Standard curves generated from target sequence cloned into a plasmid (y = −3.16 × +34.4, R^2^ = 0.997, *P* < 0.001). Dotted lines indicate points with the same Ct value (24.2).

A good relationship between Ct value and colony number was established from two series of independently prepared samples (Fig. 2a; R^2^ = 0.987, *P* < 0.001), demonstrating that artificial error variances caused by DNA extraction procedures were low. The slope of the regression line (−3.08; Fig. 2a) had a 95% confidence interval from −3.43 to −2.72 and did not differ significantly from the slope obtained from a dilution series of the template plasmid (−3.16; Fig. 2b) or the theoretical slope of −3.32. This indicates a constant DNA extraction efficiency in the range of 10^2^−10^5^ colonies and an approximately ideal efficiency of PCR amplification.

A high DNA extraction efficiency from colonies was also suggested by comparing the two standard curves as well as an estimation based on genome size. Since a single Showa colony contains 55.9 ± 7.3 cells (Average ± SE, n = 21), and the target sequence *SSL-3* is a single-copy gene in the Showa genome (MVGU01001496: 92122–92181, *B*. *braunii* strain Showa, PRJNA60039)^27^, we predict that DNA extracted from *k* colonies would contain 55.9*k* copies of the target sequence. This prediction can be validated by the plasmid standard curve (Fig. 2b). At an average number of colonies (=10^1.5^), the standard curve generated by extracting DNA from colonies (Fig. 2a) gives a predicted Ct value of 24.2 (with a 95% confidence interval from 23.8 to 24.6). The Ct value of 24.2 corresponds to 10^3.2^ copies of plasmid (Fig. 2b). Therefore, the number of target sequences per DNA extracted from one colony is estimated as 52 (=10^3.2^/10^1.5^), which agreed well with the predicted value of 55.9. Furthermore, the size of the Showa strain genome is estimated as 166.2 Mbp^28^, which indicates that 1 ng of DNA corresponds to 5,488 cells. We measured that total amount of DNA extracted from 10^5^ Showa colonies was 938 ng (SE = 19, n = 3), which corresponds to 92,000 colonies (=938*5488/55.9). This again indicates a high DNA extraction efficiency (>90%) in our standard samples.

### Amplification specificity in a natural environment

**Figure 3:**
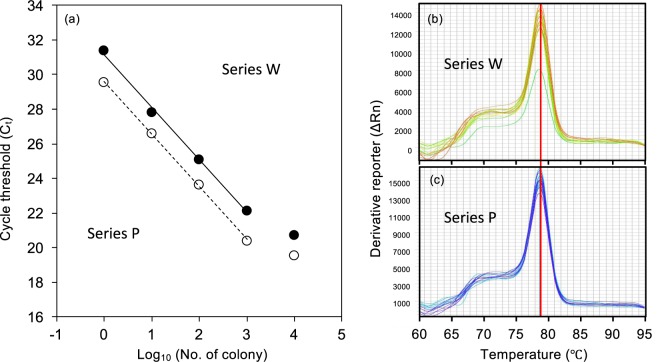
Amplification specificity of the real-time PCR assay for the *SSL-3* gene in natural pond water. (**a**) Regression line between Ct values and Log (numbers of Showa colonies) obtained from Showa culture diluted with natural pond water (○, Series P; Dotted line: y = −3.05 × +29.6, R^2^ = 0.999), compared with that obtained from Showa culture diluted with distilled water (●, Series W; Solid line: y = −3.05 × +31.2, R^2^ = 0.997). Data of 10^6^ Showa colonies are not included in the regressions. The biomass density of the pond water was 82.5 mg L^−1^ with an OD_660_ = 0.013. (**b**,**c**) Melting curve analyses of the real-time PCR products. Y-axis represents the derivative reporter (ΔRn) while x-axis represents the temperature (°C). Melting curves of the real-time PCR products of (**b**) Series W with pure Showa culture and (**c**) Series P contaminated with natural pond water.

The regression slopes of Ct values on Log (numbers of Showa colonies) did not differ significantly between sample series P (dilution by pond water) and W (dilution by distilled water; *P* > 0.1, Fig. 3a). This indicates that DNA extraction efficiency and PCR amplification were not affected by contamination with environmental DNA present in natural pond water. The melting curve analyses support the specific detection of the *SSL-3* gene in the environmental DNA samples (Fig. 3b,c). The lower intercept of the regression line of series P compared to series W (Fig. 3a) is due to the existence of wild *B*. *braunii* strains in pond water.

A decrease in DNA extraction efficiency was found in high-density samples. Samples with 10^6^ Showa colonies, which corresponds to the amount of DNA template of 10^4^ colonies (Fig. 3a), showed higher Ct values than predicted and were not included in the regressions. This can result from decreases in DNA extraction efficiency in high-density samples and/or a decrease in PCR amplification efficiency due to inhibitory effects of the extracts. Therefore, high-density samples should be diluted prior to DNA extraction and real-time PCR. As criteria needed for dilution, we measured dry weight and optical density of the samples containing 10^6^ Showa colonies: Dry weight was 18 mg for 10^6^ Showa colonies, and OD at 660 nm was 0.20 for samples with 10^6^ Showa colonies in 50 mL of distilled water. These values can be used as indicators to determine the necessity of diluting samples prior to DNA extraction, otherwise the density of *B*. *braunii* in the sample will be underestimated. Kim *et al*.^26^ also reported low DNA yields of commercial kits for DNA extraction from *B*. *braunii* cells greater than 55 mg dry weight. Such a large amount of cells will overload the filter used for collecting DNA.

### Molecular phylogeny of wild strains

**Figure 4:**
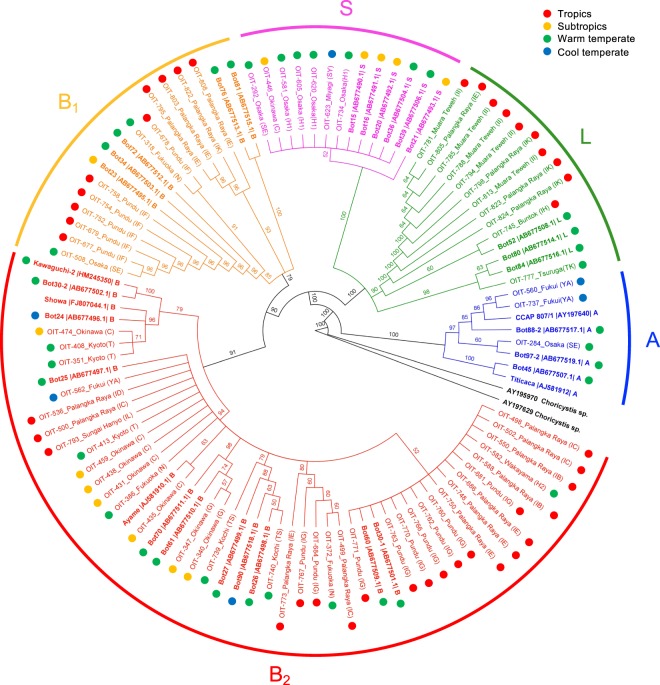
Molecular phylogenetic tree of *Botryococcus braunii* wild strains based on 18 S ribosomal RNA sequences. The tree is rooted on the branch between *Choricystis* and *Botryococcus*. Numbers around the internodes indicate bootstrap values in the Neighbourhood-joining analysis (1000 replications). Reference sequences are in bold with accession numbers and classification of chemical race (A, B, L, S). The geographic location of isolation sites is also indicated.

Figure 4 shows a molecular phylogenetic tree of *B*. *braunii* including 70 wild strains isolated from temperate to tropical ponds with reference sequences obtained from NCBI. The four chemical races (A, B, L, and S) were classified into four major clades as previously reported by Kawachi *et al*.^23^, indicating that they are genetically distinct. The chemical race S clade shows a relatively low genetic variation, indicating a recent divergence from the L clade (Fig. 4). In the B-race clade, a large sub-clade was formed with a 91% bootstrap support, which was named the B_2_ clade, and includes the standard strain Showa. Other strains in the B-race clade have a large genetic variation and did not form clear sub-clade. All were classified into the B_1_ clade, which has a 79% bootstrap support.

Our tropical strains were all classified into either the B- or L-race clade, indicating that B- and L-race strains are major components of tropical *B*. *braunii*. In the B- or L-race clade, there was no clear genetic differentiation between tropical and temperate strains, indicating a frequent gene flow over a large geographic gradient. As Kawachi *et al*.^23^ discussed, birds or winds can disperse colonies of wild *B*. *braunii*. Consequently, as Janse van Vuuren & Levanets^19^ reviewed recently, *B*. *braunii* is known to be a cosmopolitan phytoplankton species.

### Compatibility of SSL3-targeted primers to wild strains

**Figure 5:**
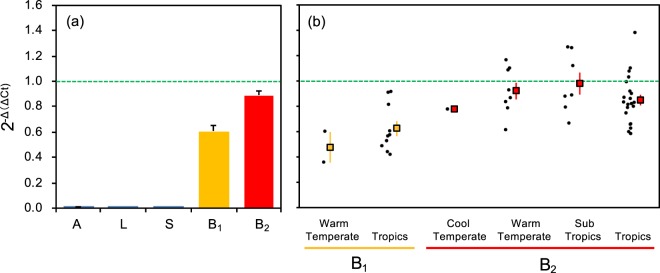
Index for PCR amplification efficiency of *SSL-3*. The index 2^−Δ(ΔCt)^ was calculated based on the Ct values of *SSL-3* and *18S* ribosomal RNA genes, and indicates the relative amplification efficiency of the *SSL-3* gene to that of standard strain Showa. (**a**) The 2^−Δ(ΔCt)^ value was obtained for the 70 wild strains, and averaged by clades. Bars indicate standard errors. (**b**) The 2^−Δ(ΔCt)^ values were averaged by climate regions where the wild strains were isolated. Filled circles indicate individual data of each strain, and rectangles are average values with standard error bars.

Table 1 summarizes the results of real-time PCR of 70 wild strains. The results were classified into 5 clades (A, L, S, B_1_, B_2_) based on the molecular phylogenetic tree. The index of amplification efficiency of the *SSL-3* gene 2^−Δ(ΔCt)^ relative to the *Showa* stain was close to zero for all strains classified as A, L, and S clades. In contrast, average 2^−Δ(ΔCt)^ values were 0.60 for wild strains in B_1_ clades and 0.89 for those in B_2_ clade (Fig. 5a). These results demonstrate that our real-time PCR method targeting *SSL-3* gene is highly specific to B-race strains, and off-target amplification of different strains (A, L, or S) would not occur. Niehaus *et al*.^21^ demonstrated that *SSL-3* was responsible for botryococcene biosynthesis in combination with another squalene synthase-like gene (*SSL-1*) and suggested that these SSL genes originated from the duplication of a progenitor squalene synthase gene. This gene duplication may occur in a progenitor of the race B clade. In fact, the biosynthesis of hydrocarbons in race A occurs through an elongation–decarboxylation route in fatty acid synthesis^12,13,29^. Thapa *et al*.^22^ identified a new gene coding squalene synthase-like protein, lycopaoctaene synthase (LOS), in race L of *B*. *braunii*, which carries out the first step in lycopadiene biosynthesis. This gene may be a good molecular marker for specific detection of race L.

**Table 1:** Summary statistics for results of real-time PCR assay using *SSL-3* and *18S* ribosomal gene primers with DNA templates of *Botryococcus braunii* strains.

Clade^†^	*N*	Ct^SSL3^		Ct^18S^		ΔCt		2^−Δ(ΔCt)^	
		Average (SE) Median	Max Min	Average (SE) Median	Max Min	Average (SE) Median	Max Min	Average (SE) Median	Max Min
A	3	31.4 (0.85) 31.6	32.8 29.9	19.1 (1.3) 19.9	20.8 16.5	12.4 (1.8) 12.8	15.2 9.1	0.0075 (0.0065) 0.0015	0.021 0.00050
L	11	33.3 (0.42) 33.4	35.1 30.4	19.7 (0.12) 19.6	20.2 19.3	13.6 (0.40) 14.0	15.5 11.1	0.0020 (0.0005) 0.0011	0.0056 0.00040
S	7	33.3 (0.73) 33.9	35.7 30.0	19.5 (0.53) 19.5	22.4 18.2	13.8 (0.89) 13.6	17.6 10.6	0.0021 (0.0009) 0.0015	0.0073 0.000095
B_1_	12	25.0 (0.28) 25.0	26.5 23.4	20.1 (0.26) 20.2	21.6 18.9	4.9 (0.15) 5.0	5.4 3.6	0.60 (0.05) 0.57	0.92 0.36
B_2_	37	25.1 (0.24) 24.8	30.3 23.2	20.8 (0.25) 20.3	26.5 19.1	4.4 (0.06) 4.4	4.9 3.0	0.89 (0.03) 0.86	1.38 0.58
Showa^§^		23.5 (0.25)		19.3 (0.27)		4.2 (0.012)		1	

^†^Clade is classified based on the molecular phylogeny of 18S ribosomal sequence.

^§^Average (SE) of Ct values with Showa DNA template (*N* = 3).

ΔCt = Ct^SSL3^ – Ct^18S^.

Δ(ΔCt) = ΔCt –ΔCt^Showa^.

Figure 5b plots individual 2^−Δ(ΔCt)^ values obtained for the 70 wild strains. The variations in 2^−Δ(ΔCt)^ values between strains within in the B-race clade were not associated with climate regions where the strains originate. Somewhat lower 2^−Δ(ΔCt)^ values from 0.3–0.6 were observed in strains classified into the B_1_ clade. As the B_1_ clade is genetically diverse and distant from the standard strain Showa, some strains may have large nucleotide sequence variations at the primer binding sites, which result in decreased amplification efficiency of the target gene. Since we focused on the exon 6 of *SSL-3* gene and designed our primers on a conserved site of the exon based on available sequences of only five strains (Supplementary Fig. S1), there might be more conserved sites for universal primers for B-race strains in the other regions of the gene. Sequencing of *SSL-3* genes of our genetically-diverse wild strains especially in the B_1_ clade will help to search for such conserved sites and to provide useful information for analyzing functionally-important regions as well as for designing universal primers for detection and quantification of B-race wild strains.

### Field testing in a natural habitat

**Figure 6:**
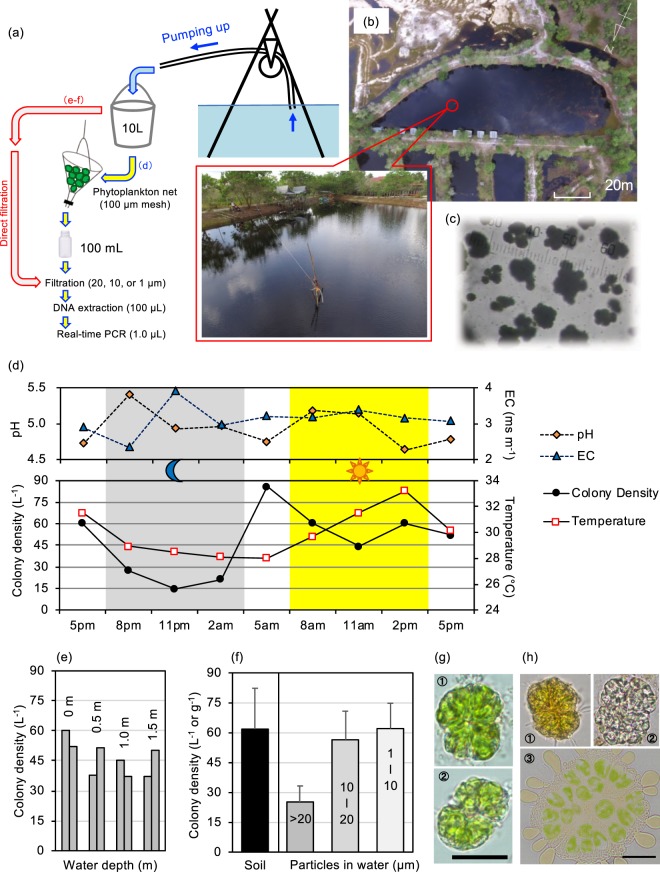
Field-testing of real-time PCR assay for detecting and quantifying wild *B*. *braunii*. (**a**) Sampling method of pond water; (**b**) Overview of the studied pond (IE), Palangka Raya, Indonesia; (**c**) Microscopic view of wild *B*. *braunii* colonies collected by a phytoplankton net; (**d**) Diurnal changes in colony density, water temperature, pH, and electrical conductivity (EC) in the surface water of the pond; (**e**) Vertical distribution of *B*. *braunii*; (**f**) Colony densities in bottom sediment (soil) and different-sized particles in water; (**g**) Small colonies found in water samples (Bar = 10 μm); (**h**) *B*. *braunii* colonies found in soil (Bar = 10 μm).

Figure 6 illustrates the field test of the real-time PCR method in a tropical pond. Our real-time PCR assay successfully quantified spatio-temporal changes in wild *B*. *braunii* abundance in the pond. A 10-L water sampling method showed a diurnal change in colony density in surface water (Fig. 6d); density decreased to half at night time. The average density at night from 8 pm to 2 am was 21.1 (L^−1^) and was significantly lower than that during the day from 8 am to 2 pm (55.0, L^−1^; ANOVA, *F*_*1*,*4*_ = 7.7, *P* < 0.01). In parallel, the average water temperature was lower at night (28.5 °C) than during the day (31.5 °C). This change in water temperature may induce vertical water circulation and result in the diurnal change in colony density in surface water. Since this diurnal change in colony density in surface water (Fig. 6d) is based on the data obtained in one day, repetitive experiments in different days and locations are necessary to confirm the observed pattern.

Quantification of the vertical distribution of *B*. *braunii* showed that this species inhabited all layers in the pond (Fig. 6e). Even in the bottom sediment, we detected *B*. *braunii* DNA (Fig. 6f), corresponding to 61.7 colonies per g soil. In fact, *B*. *braunii* colonies were easily found upon microscopic observation of the soil, some of which looked alive and contained plenty of oils (Fig. 6h). In addition, we detected *B*. *braunii* DNA in the fraction of small-sized particles <10 μm (Fig. 6f), which was confirmed by microscopic observation (Fig. 6g). The observed small-sized and sedimented colonies differ from the laboratory strain Showa cultivated in a flask, which floats up to the surface layer with a colony size generally greater than 20 μm (Fig. 1). Tanoi *et al*.^30^ reported that an iron-limitation treatment decreased colony sizes of *B*. *braunii*. The observed small and submerged colonies in the wild population may result from nutrient stresses in natural environments. The detection of *B*. *braunii* DNAs in the small size fraction (<10 μm) as well as in bottom sediments might also result from the formation of single-celled gametes for sexual reproduction or dormant cysts in the sediments. Seasonal investigations of the abundance of wild *B*. *braunii* populations using our real-time PCR assay may help elucidate its unknown ecology and cryptic life cycles in nature.

### Contributions to genetic resource exploitation and ecological studies

Our real-time PCR assay could help to efficiently exploit natural genetic resources of *B*. *braunii*. The real-time PCR method can quantify colony density of *B*. *braunii* in natural water samples in a high throughput manner (e.g., 50–100 samples per day) and help to find out high-density habitats from a large number of water samples. Subsequently, the time-consuming, microscopic isolation of wild strains can be focused on the high-density habitats. This can be an efficient strategy for wild genetic resource exploitations and might also increase a chance to isolate fast-growing strains. Because fast-growing strains are expected to increase population density, strains isolated from high-density populations are expected to be fast-growers. However, we have to note that the growth rate is not necessarily predominant factor controlling the abundance of microalgae in natural environment, since the abundance is also affected by many environmental, ecological and artificial factors (e.g., water quality, microbiome, natural enemies, and disturbance). These abiotic and biotic factors potentially affecting population density of *B*. *braunii* are also necessary to be investigated in addition to the real-time PCR quantification of *B*. *braunii* densities in natural water samples. Such efforts will elucidate underlying mechanisms of natural blooms and eventually contribute to the realization of outdoor mass cultivation of this alga for biofuel production.

## Conclusions

Based on a real-time PCR assay of a hydrocarbon biosynthetic gene, we have successfully developed a highly sensitive and specific method for detecting and quantifying the race B strain of the oil-producing microalga *Botryococcus braunii* in natural environments. This method can be widely applicable to both temperate and tropical freshwater environments and may be helpful to exploit genetic resources and to elucidate its unknown ecology and life cycles in nature.

## Methods

### Real-time PCR assay for detecting the SSL-3 gene

We designed PCR primers targeted toward the *SSL-3* gene of *B*. *braunii*. Since Niehaus *et al*.^21^ suggested that the *SSL-3* domain V is involved in functional divergence of the *SSL-3* gene from other paralogous genes (*SSL-1*, *SSL-2*), we focused on Exon 6, which encodes domain V. By nucleotide sequence alignment of the *SSL-3* gene sequences available in NCBI, we identified a conserved site on the exon 6 near domain V (Supplementary Fig. S1) and designed primers (F14, R12) targeted toward the conserved site, according to a previously published protocol^31^.

A real-time PCR assay was performed using PowerUp SYBR Green Master Mix and a StepOne instrument (Life Technologies). A 10-μL mix for each PCR run was prepared as follows: 3 μL water, 0.5 μL of each primer (0.5 μM), 1 μL DNA template, and 5 μL Fast SYBR Green Master mix. The reactions were performed using a Fast cycling mode: (1) 50 °C, 120 s; (2) 95 °C, 120 s; (3) 95 °C, 3 s; (4) 60 °C, 30 s; (5) Back to (3) 39 times, (6) 95 °C, 15 s; (7) 60 °C, 60 s; (8) 95 °C, 15 s). Average Ct values of two or three repetitions were obtained and used for analyses.

### Standard curve generation with Showa strain

We prepared DNA templates for the standard curve of the real-time PCR assay using Showa strain (race B). The Showa strain was originally isolated in a greenhouse at the University of California, Berkeley^32^ and was distributed to our laboratory. We cultivated the Showa strain in a 1-L reactor with AF-6 medium at 27 °C, with a photosynthetic photon flux density (PPFD) of 100 μmol·s^−1^·m^−2^ (14 h per day) and 3% CO_2_-bubbling. We diluted an aliquot of the Showa culture to make a 50-mL sample containing 10^6^ Showa colonies. The sample was diluted sequentially 10 times with distilled water to make two series of 50-mL samples from 10^5^ to 10^2^ colonies. The 50-mL samples were filtered individually with a 10-μm membrane filters (JCWP04700, Merck), and the filters were frozen by liquid nitrogen and disrupted using a multi-bead shocker instrument (Yasui Kikai, Japan) at 3000 rpm for 30 sec. DNA was extracted from the disrupted filter using a NucleoSpin Plant II (Macherey-Nagel, Germany) according to the manufacturer’s protocol, and a 100-μL DNA solution was obtained from each sample. Real-time PCR assay was performed as described above using 1 μL of the DNA solution as a template. The Ct values were plotted against Log_10_-transformed colony densities per μL of DNA solution to make a standard curve. We also generated a standard reference curve using the target sequence cloned into a plasmid. The target sequence amplified from the DNA of Showa strain was cloned into pMD20 plasmid vector (TaKaRa, Japan), and the plasmids were extracted using NucleoSpin Plasmid EasyPure (Macherey-Nagel, Germany), following the manufacturer’s protocols. The DNA concentrations of the plasmid sample were determined using a fluorometer (Qubit 2.0, Life Technologies). Average cell number per colony was estimated by counting cells of Showa colonies (n = 21) flattened by cover glass (Fig. 1c).

### Amplification specificity in a natural environment

To test the PCR amplification specificity in a natural environment, we prepared a series of standard samples diluted in pond water. A 1-L sample of natural pond water was taken from a pond around Osaka castle, Osaka city, Japan in August 2017 using a plankton net with a 100-μm mesh. Microscopic observations confirmed the presence of several microalgal species, including *B*. *braunii*, in the pond. Pond water was used to dilute an aliquot of Showa culture to prepare a 50-mL sample with 10^6^ Showa colonies (Supplementary Fig. S2). The sample was diluted 10X sequentially with pond water to make samples containing 10^5^ to 10^2^ Showa colonies (Series P). For comparison, sample preparation was repeated using distilled water instead of pond water (Series W). The samples were filtered with membrane filters and DNA was extracted from the filters as described above. Real-time PCR assays were performed using the extracted DNAs as templates and standard curve relationships between Ct and colony density were compared between the two series.

### Isolation of wild strains

**Figure 7:**
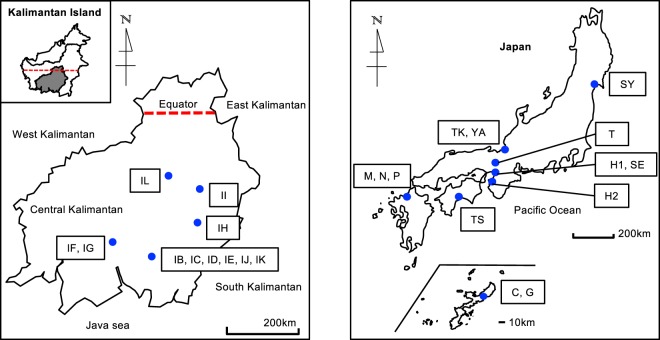
Map of the sampling locations of wild *Botryococcus braunii* at Central Kalimantan, Indonesia, and Japan. Palangka Raya (Pond IB, IC, ID, IE, IJ, IK); Pundu (IF, IG); Buntok (IH); Muara Teweh (II); Sungei Hanyo (IL); Okinawa (C, G); Fukuoka (M, N, P); Kochi (TS); Fukui (TK, YA); Wakayama (H2); Osaka (H1, SE); Kyoto (T); Miyagi (SY). A total of 70 wild strains were isolated from the 24 ponds distributed from tropics (Indonesia), subtropics (Okinawa), warm temperate (Fukuoka, Kochi, Fukui TK, Wakayama, Osaka, Kyoto), and cool temperate regions (Miyagi, Fukui YA).

Seventy wild *B*. *braunii* strains were isolated from tropical to temperate freshwater environments (Fig. 7). Microalgae in surface water were collected using a plankton net with a 100-μm mesh. A single colony of *B*. *braunii* was isolated by micropipette, transferred to a glass tube containing AF-6 medium, and incubated at 25 °C with a 12-h light/12-h dark cycle with fluorescence lamps with a PPFD of 100 μmol m^−2^ s^−1^. After one month, colonies were transferred to 30-mL culture bottles and incubated for an additional two months. Colonies were then collected with a 10-μm filter membrane, and DNA was extracted from the filter. DNA concentration was determined by using a fluorometer (Qubit 2.0, Life Technologies) and was diluted to 1 ng μL^−1^.

### 18S ribosomal sequencing and molecular phylogeny

18 S ribosomal RNA sequences were determined for the 70 isolated wild strains to estimate phylogenetic relationships with previously described strains^23^ and classify them into different chemical races. Either specific primers (63 F & 1818R)^23^ or universal primers (EukF1 & EukR1)^33^ were used to amplify 18 S ribosomal RNA sequences (Supplementary Table S1) by EmeraldAmp PCR Mater Mix (TaKaRa Bio, Japan) including 0.2 μM of primers and 1 ng of DNA template with the following thermal cycler program: 2 min at 95 °C; 34 cycles of 95 °C/30 s, 55 °C/30 s, 72 °C/100 s; and 5 min at 72 °C. The PCR products were directly sequenced. These sequences were aligned using ClustalW with additional reference sequences obtained from NCBI. Neighbour-joining (NJ) analysis with the Tamura-Nei model of genetic distance was performed using the sequence of *Choricystis* sp., the closest species to *Botryococcus* among the members of Trebouxiophyceae^23^, as an outgroup. Bootstrap values of 1000 replicates were obtained using Geneious R11.

### Compatibility of SSL3-gene primers with wild strains

To assess the wide applicability of our real-time PCR method for detecting and quantifying wild *B*. *braunii* in natural environments, we tested the efficiency of our PCR amplification for 70 wild strains based on the theory of relative quantification with an internal control gene^34^. First, real-time PCR assays were performed using the *SSL-3* gene primers and DNA templates of wild strains, and Ct values (Ct^SSL3^) were obtained. Next, as an internal control, we designed primers targeted toward a conserved region of 18S ribosomal RNA sequences of the 70 wild strains: Bot18S_qF1 and Bot18S_qR1 (Supplementary Table S1) and real-time PCR assays were performed using the 18S primers and the same DNA templates of wild strains to obtain Ct values (Ct^18S^). The differences of the Ct values, ΔCt (= Ct^SSL3^ − Ct^18S^) were calculated for each strain. The ΔCt is expected to increase if the *SSL-3* primers did not match the template DNA. The Δ(ΔCt) value was calculated as Δ(ΔCt) = ΔCt^wild strain^ − ΔCt ^Showa^. The Δ(ΔCt) is expected to be zero when PCR amplification efficiencies of target and internal control genes are identical to those of the Showa strain, and would increase as the PCR amplification efficiency of the target sequence decreases. The relative level of PCR amplification efficiency was estimated as 2^−Δ(ΔCt)^.

### Field testing in a natural habitat

In pond IE, Palangka Raya, Kalimantan Island, Indonesia, we performed field testing of the real-time PCR assay. Pond IE was created more than 20 years ago at an ex-mining site and has been utilized as a recreational pond for boating and fishing. The pond naturally holds water throughout the year. We first found a natural *B*. *braunii* population in the pond in August 2015. In September 2017, 10-L surface water samples were taken from the centre of the pond every three hours to estimate colony density and its diurnal changes in the pond. The sample was filtered by a phytoplankton net with a 100 μm mesh and was concentrated to 100 mL. The 100-mL sample was filtered under reduced pressure with a dual Kimwipe paper, then the paper was freeze-dried, a quarter of which was used for DNA extraction by the previously described method. A 1-μL DNA solution was used for the real-time PCR assay to estimate the number of *B*. *braunii* colonies (L^−1^) in the surface water. Water temperature, pH, and electrical conductivity (EC) were also determined at sampling time. Once per day (at 5 p.m.), we also obtained additional 10-L samples from depths of 50, 100, and 150 cm to estimate vertical changes in colony densities. The approximate water depth at the sampling point was 150–200 cm. The following year, in October 2018, 5-L surface water samples were taken from three points along the shore of the pond to estimate the distribution of *B*. *braunii* in different-sized particles in the water. The sampled water was filtered with 20-μm, 10-μm, and 1-μm membrane filters sequentially, then DNA was extracted from the filters as described above. We also manually sampled bottom sediments in the centre of the pond, and DNA was extracted from 0.5-g soil samples using ISOIL for bead beating (NIPPON GENE, Japan) according to the manufacturer’s protocol. Six replicates were used for the soil DNA extraction. Colony density (L^−1^ water or g^−1^ soil) was calculated from the Ct value and the volume of filtered water or the weight of soil.

## Supplementary information

Supplementary file

## Data Availability

18S ribosomal RNA sequences of the 70 wild *Botryococcus braunii* strains are available from DDBJ (Accession no. LC468958-LC469027).
