# Supplementary material for: Detection of the oil-producing microalga Botryococcus braunii in natural freshwater environments by targeting the hydrocarbon biosynthesis gene SSL-3
Source: Sci Rep. 2019 Nov 18;9:16974. doi: 10.1038/s41598-019-53619-y (PMC6861321; doi:10.1038/s41598-019-53619-y)
Supplement: Supplementary file 1 — Supplementary file [file 41598_2019_53619_MOESM1_ESM.pdf]

Supplementary Information

Scientific Reports

Detection of the oil-producing microalga *Botryococcus braunii* in natural freshwater environments by targeting the hydrocarbon biosynthesis gene *SSL-3*

Kotaro Hirano, Takuya Hara, Ardianor, Rudy Agung Nugroho, Hendrik Segah, Naru Takayama, Gumiri Sulmin, Yukio Komai, Shigeru Okada, Koji Kawamura*

*Corresponding author

E-mail: koji.kawamura@oit.ac.jp

Figure S1. Alignment of the nucleotide sequences of exon 6 of *squalene synthase like-3* genes (*SSL-3*) for five strains of *Botryococcus braunii* race B. Partial cDNA sequences of Showa (HQ585060), Indian strains (KR673392, KR673396, KR673400), and Ayame (KU248135) are aligned.

Figure S2. Sample preparation for testing PCR amplification specificity in a natural environment. Two series of samples containing 10^6 to 10^2 Showa colonies were made. One series (P) was diluted using pond water, whereas in another series (W), distilled water was used for dilution.

Table S1. Primer list.

Table S2. List of 70 wild *Botryococcus braunii* strains. Clades were determined by generating a molecular phylogenetic tree from 18S ribosomal RNA sequences.

Supplementary Figure S1

Hirano et al. *Scientific Reports*

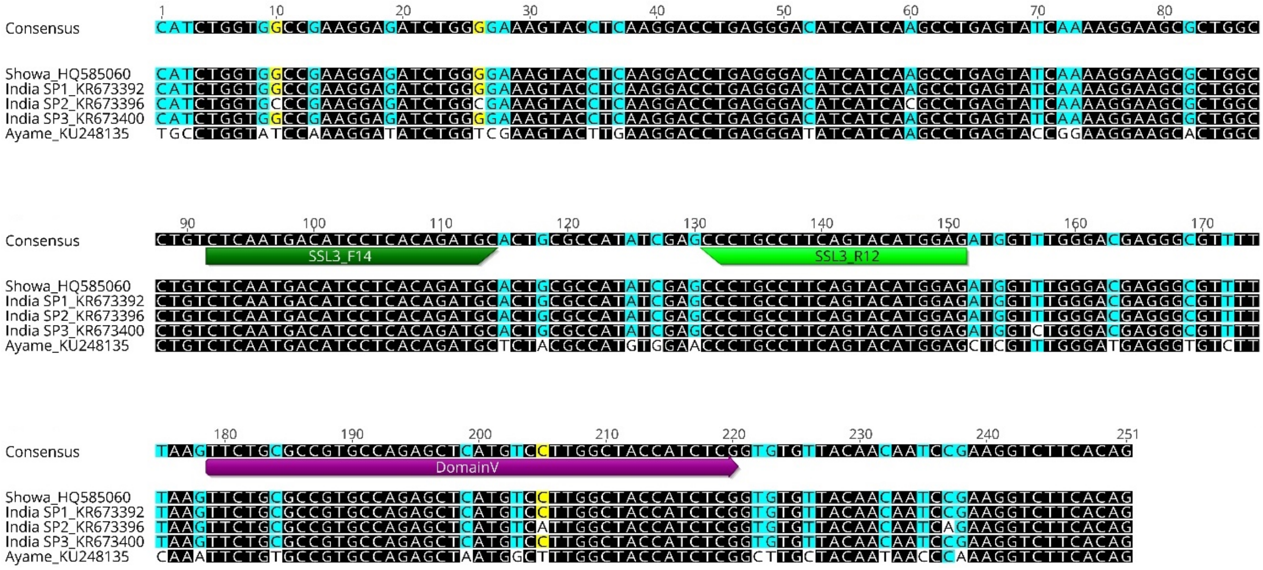

Figure S1. Alignment of the nucleotide sequences of exon 6 of *squalene synthase like-3* genes (*SSL-3*) for five strains of *Botryococcus braunii* race B. Partial cDNA sequences of Showa (HQ585060), Indian strains (KR673392, KR673396, KR673400), and Ayame (KU248135) are aligned.

Supplementary Figure S2

Hirano et al. *Scientific Reports*

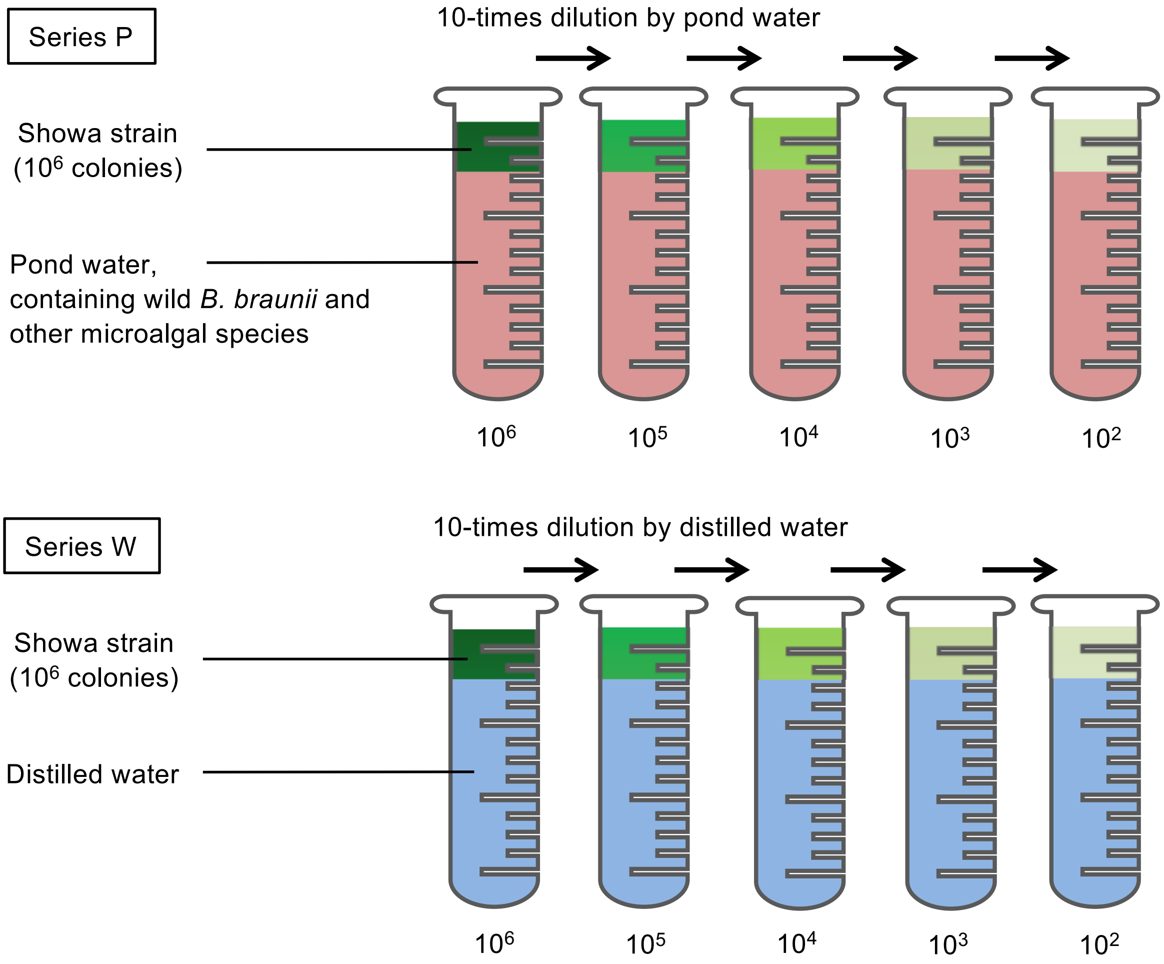

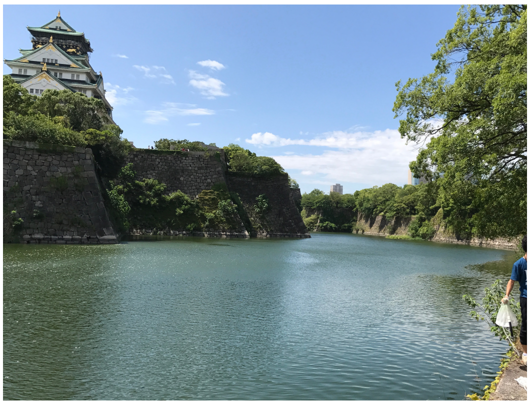

Pond around Osaka castle, Japan

Figure S2. Sample preparation for testing PCR amplification specificity in a natural environment. Two series of samples containing 10^6 to 10^2 Showa colonies were made. One series (P) was diluted using pond water, whereas in another series (W), distilled water was used for dilution.

Supplementary Table S1

Hirano et al. *Scientific Reports*

Table S1. Primer list.

Primer name	Target	Sequence (5'-3')	Product size (bp)	Ref
SSL3_F14	SSL-3	CTCAATGACATCCTCACAGATGC	60	1)
SSL3_R12		CTCCATGTACTGAAGGCAGGG		
Bot18S_63F	18S rRNA	ACGCTTGTCTCAAAGATTA	1747	2)
Bot18S_1818R		ACGGAAACCTTGTTACGA		
18S_EukF1	18S rRNA	ACCTGGTTGATCCTGCCAG	1795	3)
18S_EukR1		TGATCCTTCYGCAGGTTCAC		
Bot18S_qF1	18S rRNA	CCCTCCAGCACCTTATGAGAA	67	1)
Bot18S_qR1		TTTCAGCCTTGCGACCATACT		

1) This study

2) Kawachi et al (2012) Algal Res. **1**: 114-119.

3) Moon et al (2000) Limnol. Oceanogr. **45**: 98-109.

Supplementary Table S2

Hirano et al. *Scientific Reports*

Table S2. List of 70 wild Botryococcus braunii strains. Clade was determined by molecular phylogenetic tree of 18S ribosomal sequences.

ID	Sampling site	Climate	Sampling date	Clade of molecular phylogeny	Accession no. 18S ribosomal RNA sequence
OIT-284	Osaka, Japan (Pond SE)	Warm Temperate	22-Jan-14	A	LC468958
OIT-292	Osaka, Japan (Pond SE)	Warm Temperate	17-Jan-14	S	LC468959
OIT-318	Fukuoka, Japan (Pond N)	Warm Temperate	08-May-15	B1	LC468960
OIT-340	Okinawa, Japan (Pond G)	Subtropics	17-Mar-15	B2	LC468961
OIT-347	Okinawa, Japan (Pond G)	Subtropics	17-Mar-15	B2	LC468962
OIT-351	Kyoto, Japan (Pond T)	Warm Temperate	07-May-15	B2	LC468963
OIT-372	Fukuoka, Japan (Pond N)	Warm Temperate	10-Apr-15	B2	LC468964
OIT-386	Fukuoka, Japan (Pond N)	Warm Temperate	10-Apr-15	B2	LC468965
OIT-408	Kyoto, Japan (Pond T)	Warm Temperate	02-Jun-15	B2	LC468966
OIT-413	Kyoto, Japan (Pond T)	Warm Temperate	02-Jun-15	B2	LC468967
OIT-431	Okinawa, Japan (Pond C)	Subtropics	05-Mar-15	B2	LC468968
OIT-435	Okinawa, Japan (Pond C)	Subtropics	05-Mar-15	B2	LC468969
OIT-438	Okinawa, Japan (Pond C)	Subtropics	05-Mar-15	B2	LC468970
OIT-446	Okinawa, Japan (Pond C)	Subtropics	05-Mar-15	S	LC468971
OIT-459	Okinawa, Japan (Pond C)	Subtropics	05-Mar-15	B2	LC468972
OIT-474	Okinawa, Japan (Pond C)	Subtropics	09-Mar-15	B2	LC468973
OIT-498	Palangka Raya, Indonesia (Pond IC)	Tropics	08-Sep-15	B2	LC468974
OIT-499	Palangka Raya, Indonesia (Pond IC)	Tropics	08-Sep-15	B2	LC468975
OIT-500	Palangka Raya, Indonesia (Pond IC)	Tropics	20-Sep-15	B2	LC468976
OIT-502	Palangka Raya, Indonesia (Pond IC)	Tropics	09-Sep-15	B2	LC468977
OIT-508	Osaka, Japan (Pond SE)	Warm Temperate	15-Feb-16	B1	LC468978
OIT-536	Palangka Raya, Indonesia (Pond ID)	Tropics	02-Mar-16	B2	LC468979
OIT-550	Palangka Raya, Indonesia (Pond IB)	Tropics	13-Apr-16	B2	LC468980
OIT-560	Fukui, Japan (Pond YA)	Cool Temperate	12-May-16	A	LC468981
OIT-562	Fukui, Japan (Pond YA)	Cool Temperate	18-May-16	B2	LC468982
OIT-581	Osaka, Japan (Pond H1)	Warm Temperate	13-May-16	S	LC468983
OIT-582	Wakayama, Japan (Pond H2)	Warm Temperate	16-May-16	B2	LC468984
OIT-588	Palangka Raya, Indonesia (Pond IB)	Tropics	18-May-16	B2	LC468985
OIT-605	Osaka, Japan (Pond H1)	Warm Temperate	13-May-16	S	LC468986
OIT-620	Osaka, Japan (Pond H1)	Warm Temperate	27-Sep-16	S	LC468987
OIT-623	Miyagi, Japan (Pond SY)	Cool Temperate	17-Oct-16	S	LC468988
OIT-677	Pundu, Indonesia (Pond IF)	Tropics	21-Mar-17	B1	LC468989
OIT-678	Pundu, Indonesia (Pond IF)	Tropics	21-Mar-17	B1	LC468990
OIT-679	Pundu, Indonesia (Pond IF)	Tropics	21-Mar-17	B1	LC468991
OIT-681	Pundu, Indonesia (Pond IG)	Tropics	21-Mar-17	B2	LC468992
OIT-684	Pundu, Indonesia (Pond IG)	Tropics	21-Mar-17	B2	LC468993
OIT-686	Palangka Raya, Indonesia (Pond IE)	Tropics	27-Mar-17	B2	LC468994
OIT-734	Osaka, Japan (Pond H1)	Warm Temperate	31-May-17	S	LC468995
OIT-737	Fukui, Japan (Pond YA)	Cool Temperate	31-May-17	A	LC468996
OIT-739	Kochi, Japan (Pond TS)	Warm Temperate	31-May-17	B2	LC468997
OIT-740	Kochi, Japan (Pond TS)	Warm Temperate	31-May-17	B2	LC468998
OIT-745	Buntok, Indonesia (Pond IH)	Tropics	01-Jun-17	L	LC468999
OIT-748	Palangka Raya, Indonesia (Pond IE)	Tropics	01-Jun-17	B2	LC469000
OIT-750	Palangka Raya, Indonesia (Pond IE)	Tropics	01-Jun-17	B2	LC469001
OIT-752	Pundu, Indonesia (Pond IF)	Tropics	01-Jun-17	B1	LC469002
OIT-754	Pundu, Indonesia (Pond IF)	Tropics	01-Jun-17	B1	LC469003
OIT-758	Pundu, Indonesia (Pond IF)	Tropics	01-Jun-17	B1	LC469004
OIT-760	Pundu, Indonesia (Pond IG)	Tropics	01-Jun-17	B2	LC469005
OIT-762	Pundu, Indonesia (Pond IG)	Tropics	01-Jun-17	B2	LC469006
OIT-763	Pundu, Indonesia (Pond IG)	Tropics	01-Jun-17	B2	LC469007
OIT-766	Pundu, Indonesia (Pond IG)	Tropics	01-Jun-17	B2	LC469008
OIT-767	Pundu, Indonesia (Pond IG)	Tropics	01-Jun-17	B2	LC469009
OIT-770	Pundu, Indonesia (Pond IG)	Tropics	01-Jun-17	B2	LC469010
OIT-771	Pundu, Indonesia (Pond IG)	Tropics	01-Jun-17	B2	LC469011
OIT-773	Palangka Raya, Indonesia (Pond IE)	Tropics	01-Jun-17	B2	LC469012
OIT-777	Fukui, Japan (Pond TK)	Warm Temperate	06-Sep-16	L	LC469013
OIT-781	Muara Teweh, Indonesia (Pond II)	Tropics	04-Oct-17	L	LC469014
OIT-785	Muara Teweh, Indonesia (Pond II)	Tropics	04-Oct-17	L	LC469015
OIT-788	Muara Teweh, Indonesia (Pond II)	Tropics	04-Oct-17	L	LC469016
OIT-793	Sungai Hanyo, Indonesia (Pond IL)	Tropics	04-Oct-17	B2	LC469017
OIT-794	Muara Teweh, Indonesia (Pond II)	Tropics	05-Oct-17	L	LC469018
OIT-795	Palangka Raya, Indonesia (Pond IE)	Tropics	05-Oct-17	B1	LC469019
OIT-798	Palangka Raya, Indonesia (Pond IK)	Tropics	06-Oct-17	L	LC469020
OIT-803	Palangka Raya, Indonesia (Pond IE)	Tropics	11-Oct-17	B1	LC469021
OIT-805	Palangka Raya, Indonesia (Pond IE)	Tropics	17-Oct-17	L	LC469022
OIT-808	Palangka Raya, Indonesia (Pond IE)	Tropics	17-Oct-17	B1	LC469023
OIT-813	Muara Teweh, Indonesia (Pond II)	Tropics	17-Oct-17	L	LC469024
OIT-822	Palangka Raya, Indonesia (Pond IK)	Tropics	22-Feb-18	B1	LC469025
OIT-823	Palangka Raya, Indonesia (Pond IK)	Tropics	22-Feb-18	L	LC469026
OIT-824	Palangka Raya, Indonesia (Pond IK)	Tropics	22-Feb-18	L	LC469027
